# Some Concerns from a Radiological Point of View. Comment on Huang et al. Outcomes of Conversion Surgery for Metastatic Gastric Cancer Compared with In-Front Surgery Plus Palliative Chemotherapy or In-Front Surgery Alone. *J. Pers. Med.* 2022, *12*, 555

**DOI:** 10.3390/jpm12071061

**Published:** 2022-06-29

**Authors:** Maria Antonietta Mazzei, Giulio Bagnacci, Armando Perrella, Nunzia Di Meglio, Stefania Angela Piccioni, Francesco Bloise, Daniele Marrelli, Carlo Milandri, Gianni Mura

**Affiliations:** 1 Unit of Diagnostic Imaging, Department of Medical, Surgical and Neuro Sciences and of Radiological Sciences, University of Siena, Azienda Ospedaliero-Universitaria Senese, 53100 Siena, Italy; mariaantonietta.mazzei@unisi.it (M.A.M.); dimeglionunzia@gmail.com (N.D.M.); 2 Unit of General Surgery and Surgical Oncology, Department of Medicine Surgery and Neurosciences, University of Siena, 53100 Siena, Italy; stefipiccioni@gmail.com (S.A.P.); daniele.marrelli@unisi.it (D.M.); 3 Department of Oncology, San Donato Hospital, 52100 Arezzo, Italy; francesco.bloise@uslsudest.toscana.it (F.B.); carlo.milandri@uslsudest.toscana.it (C.M.); 4 Department of Surgery, San Donato Hospital, 52100 Arezzo, Italy; gianni.mura@uslsudest.toscana.it

We read, with great interest, the article by Huang Ruo-Yi and colleagues entitled “Outcomes of Conversion Surgery for Metastatic Gastric Cancer Compared with In-Front Surgery Plus Palliative Chemotherapy or In-Front Surgery Alone”, published on 1 April 2022 [1].

The study demonstrated the survival benefits of conversion surgery in patients with metastatic gastric cancer (mGC) when compared to in-front surgery plus palliative chemotherapy (PTC) or in-front surgery alone (median OS 23.4 vs. 13.7 vs. 5.6 months, respectively); however, the study was conducted in a small cohort of patients (182 enrolled patients: 25–13.7%—conversion surgery patients; 101–55.5%—in-front surgery plus PCT patients; and 56–30.8%—in-front surgery alone patients) and with a huge variety of chemotherapy regimens and time durations (the median duration of chemotherapy before conversion surgery was 5.9, with a range of 2.3–21.7 months).

Regarding the conversion surgery group (25 patients), the authors also stated that:

(a) Patients who underwent conversion surgery with downstaging (stage I–III vs. stage IV) had a better prognosis than those without downstaging;
(b) Patients without distant node metastasis had better a prognosis than those with distant node metastasis (*p* = 0.021); in contrast, there were no significant differences in patient outcomes in terms of peritoneal/omental (*p* = 0.418), liver (*p* = 0.093), or ovarian metastasis (*p* = 0.488).

Considering that in the conversion surgery group: (i) distant nodal metastases were identified in 12 patients (48.0%), peritoneal/omental metastases in 9 patients (36.0%), liver metastases in 5 patients (20.0%), and ovarian metastases in 3 (12.0%) patients; (ii) downstaging (pathological stage I–III) was noted in 15 (60%) and non-downstaging in 10 (40%) patients; and finally, (iii) tumor response was defined using the Response Evaluation Criteria in Solid Tumors (RECIST), some concerns arise from a radiological point of view. 

As is known, the use of the RECIST alone might bias the evaluation of treatment response in gastric cancer, because the response to therapy cannot be evaluated in patients without a measurable (target) metastatic lesion: ≥10 mm for a hematogenous lesion in their longest diameter or ≥15 mm for metastatic lymph node in their short axis, since both the primary lesion and peritoneal dissemination are considered non-measurable lesions for RECIST [2,3,4].

That being said, if the downstaging was obtained in conversion surgery patients with distant nodal metastases, we suppose these patients should all have metastatic nodes with a short-axis diameter ≥15 mm at staging CT. Conversely, since peritoneal metastases are not considered target lesions for RECIST, how do the authors evaluate downstaging in patients with peritoneal/omental metastases (if there were any among them)?

In our case studies of mGC patients, distant node metastases were identified in 32/74 patients, and they were the only metastatic site in 7/74 patients. The median short axis of metastatic distant nodes at staging CT was 14.5 ± 5.9 mm, and in only 11/32 patients (34%) did distant nodes have a short axis ≥15 mm, thus being eligible as target lesions for RECIST 1.1 evaluation (moreover, only 28/32 presented with a short axis >10 mm). The unsuitability of those criteria for lymph nodes has been demonstrated for some other neoplasms [5].

A separate note deserves to be made for peritoneal metastases. In our case study, peritoneal metastases were identified in 58/74 patients and in 17/74 patients as the only metastatic site. However, none of the patients showed peritoneal “measurable” lesions at staging CT.

Regarding response evaluation, only a small number of our patients have undergone restaging CT (35 patients) at present (Table 1). The RECIST 1.1 criteria state that to assess peritoneal progression of the disease, “unequivocal progression” of the non-target lesion should be present, even if the criteria to define it remain unclear. Considering our population, radiological peritoneal progression was present in 12/35 patients, but only 3 of them can be considered “PD” with strict application of RECIST1.1 criteria. In view of the high number of doubtful cases, a revision of the response evaluation criteria focusing on peritoneal metastasis should be encouraged.

This comment is not supposed to belittle the author’s observations, which are definitely impressive and deserve to be disseminated. On the contrary, considering the strong message regarding the better prognosis of patients with downstaging—a clinical condition assessable and measurable only with imaging—this comment intends to emphasize that the evaluation of the response to therapy in mGC patients requires dedicated imaging criteria to better predict the prognosis and guide multimodal treatments.

## Figures and Tables

**Table 1:** An outline of our patients would be classified considering RECIST 1.1 with or without adding the peritoneal assessment. Note that 9 patients (25%) would have been reclassified.

		RECIST1.1 and Peritoneum		
		PD	SD	PR
RECIST 1.1	PD	3	0	0
	SD	7	10	0
	PR	2	0	13

## Data Availability

The data presented in this study are available on request from the corresponding author.
